# Altered hemispheric asymmetry of attentional networks in patients with pituitary adenoma: an event-related potential study

**DOI:** 10.3389/fnins.2023.1198409

**Published:** 2023-05-11

**Authors:** Shuochen Wang, Zhenghao Fu, Yuanyi Sun, Meng Zhang, Aobo Chen, Chenglong Cao, Jian Song

**Affiliations:** ^1^The First School of Clinical Medicine, Southern Medical University, Guangzhou, Guangdong, China; ^2^Department of Neurosurgery, The General Hospital of Central Theater Command, Wuhan, Hubei, China; ^3^Department of Neurosurgery, The First Affiliated Hospital of University of Science and Technology of China, Hefei, Anhui, China

**Keywords:** event-related potentials, lateralized attention network test, hemispheric asymmetry, alerting, orienting, executive control, pituitary adenoma

## Abstract

**Background:**

Emerging evidence has been reported of attentional dysfunction in pituitary adenoma patients. However, the effect of pituitary adenomas on lateralized attention network efficiency remained to be clear. Thus, the present study aimed to investigate the impairment of lateralized attention networks in patients with pituitary adenoma.

**Methods:**

Eighteen pituitary adenoma patients (PA group) and 20 healthy controls (HCs) were included in this study. Both behavioral results and event-related potentials (ERPs) were acquired while subjects performed the Lateralized Attention Network Test (LANT).

**Results:**

Behavioral performances indicated the PA group had a slower reaction time and a similar error rate relative to the HCs group. Meanwhile, significantly increased executive control network efficiency suggested the dysfunction of inhibition control in PA patients. Regarding ERP results, there were no group differences in the alerting and orienting networks. The target-related P3 was significantly reduced in the PA group, suggesting an impairment of executive control function and attentional resources allocation. Moreover, the mean amplitude of P3 was significantly lateralized to the right hemisphere, and interacted with the visual field, exhibiting that the right hemisphere dominated the bilateral visual field, whereas the left hemisphere dominated the left visual field. In the specific high-conflict condition, the pattern of hemispheric asymmetry in the PA group was altered due to a mixed effect resulting from the compensatory recruitment of attentional resources in the left central parietal area and the destructive effects of hyperprolactinemia.

**Conclusion:**

These findings suggested that, in the lateralized condition, the decreased P3 in the right central parietal area and the diminished hemispheric asymmetry under high conflict load, may serve as the potential biomarkers of attentional dysfunction in patients with pituitary adenoma.

## 1. Introduction

Pituitary adenoma (PA) is one of the common benign intracranial tumors of the central nervous system, accounting for almost 15% of all cases (Hauser et al., 2019; Melmed, 2020). Emerging studies have established cognitive impairments in executive control, attention, and working memory both before and after surgery in patients with pituitary adenoma (Psaras et al., 2011; Butterbrod et al., 2019; Pertichetti et al., 2020), but the underlying neurophysiological mechanisms remain unclear. The physical compression, treatment strategy, surgical approach, and especially abnormal hormone levels may be responsible for the impairment of cognitive function (Peace et al., 1997, 1998; de Oliveira et al., 2008; Tooze et al., 2009; Tooze and Sheehan, 2018). In Yao’s opinion (Yao et al., 2017), prolactinoma patients exhibited decreased gray matter volume (GMV) in the left hippocampus, left orbitofrontal cortex, right middle frontal cortex, and right inferior frontal cortex, providing seminal evidence for deficits in verbal memory and executive control in patients with prolactinoma. The dysfunction of attention and inhibition control has been extensively examined in patients with pituitary adenomas based on neuropsychological scales and behavioral outcomes (Müssig et al., 2011; Pertichetti et al., 2020). To our knowledge, however, there have been few studies investigating the lateralized attentional networks in PA patients systematically.

According to the classical attention network theory, proposed by Posner and colleagues (Posner and Petersen, 1990; Petersen and Posner, 2012; Posner et al., 2019), the attention network has been divided into alerting, orienting, and executive control networks: Alerting refers to the state of obtaining and maintaining vigilance to upcoming information. This network may be associated with the right hemisphere (RH), frontal, parietal, and thalamic regions, and influenced by the norepinephrinergic system. Orienting network is for selecting specific information from the environment by focusing on one modality or location. Orienting is related to the cholinergic system and is associated with the frontal eye field (FEF), intraparietal sulcus (IPS), and other areas. Executive control network plays a major role in monitoring and resolving task-related conflicts, including error decision-making, and planning. This network primarily involved the dorsal anterior cingulate cortex and the lateral prefrontal cortex and corresponded to the dopaminergic system.

Despite their bilateral distribution, studies have shown that attentional functions might be dominated in the right hemisphere, particularly in the right parietal area (Mesulam, 1999; Brooks et al., 2014). Furthermore, for visuospatial attention, attending to one side of the visual stimuli typically corresponds to the activation of contralateral parietal areas. Based on the above findings and the ANT paradigm, Greene and colleagues proposed the LANT paradigm to assess the hemispheric asymmetry in each attention network as well as the attentional volume of each hemisphere (Greene et al., 2008). The LANT paradigm revealed the hemispheric lateralization of each attention network by rotating the original up and down target stimuli by 90° and presenting to the left and right visual fields (LVF, RVF). Previous studies of attentional networks indicated that multiple disorders, including mild traumatic brain injury (Chen et al., 2021) and attention deficit hyperactivity disorder (ADHD) (Adólfsdóttir et al., 2008; Lundervold et al., 2011), have the potential to impair attentional networks. Apart from a few studies related to stroke (Russell-Giller et al., 2021) and cerebral small vessel disease (Cao et al., 2020), to the best of our knowledge, systematic and comprehensive investigations based on the theory of the lateralized attention network in patients with pituitary adenoma were limited.

Referring to the comparable Attention Network Test (ANT) studies (Fan et al., 2002, 2005; Neuhaus et al., 2010; Williams et al., 2016), the cue-N1 component was associated with alerting and orienting networks. N1 was defined as an early visual attention component that appeared 150–250 ms after the cue stimulus and was distributed in the parietal and occipital regions. N1 was considered an early visual processing of stimulus properties, and the amplitude increased when the visual stimulus appeared in the attended spatial location. Furthermore, N1 also reflected the facilitation of early preattentive processing (Kaufman et al., 2016). Target-P3 components were associated with the executive control network and typically appeared around 250-500 ms after target presentation. P3 originated in the anterior cingulate gyrus and was located in the central parietal region, which mirrored the response inhibition process and attentional resource allocation (Polich, 2004, 2007).

In summary, in the present study, the LANT paradigm and ERPs were combined to investigate the hemispheric lateralization of attention networks in patients with pituitary adenoma. We hypothesized that (1) PA patients have significantly decreased behavioral and ERP results in attention networks; (2) Attention networks might be exhibited hemispheric asymmetry in two groups; (3) The pattern of hemispheric asymmetry in PA patients might be different from that in HCs; and (4) The serum prolactin (PRL) level may impair attention networks in patients with pituitary adenoma.

## 2. Materials and methods

### 2.1. Participants

Twenty patients with pituitary adenoma and 25 healthy adults matched for gender, age, and education were recruited from the General Hospital of Central Theater Command. Inclusion criteria for the PA group were: (1) Age: 16–55 years old; (2) Right-handedness; (3) Education: more than 6 years; (4) Tumor size: less than 30 mm; (5) Vision: normal or corrected visual acuity and visual field (VF); and (6) Pathological diagnosed with pituitary adenoma. Exclusion criteria for the PA group were: (1) Recurrent pituitary adenoma or pituitary apoplexy and (2) Had taken dopaminergic inhibitors such as bromocriptine or radiation therapy such as gamma knife before surgery. Common exclusion criteria for both groups were as follows: (1) Had taken neurological and psychotropic drugs such as dipipanone; (2) History of drug or alcohol abuse in the past 3 months before surgery; (5) Female subjects who were menstruating. Informed consent was obtained from all subjects, and the study was approved by the ethics committee of the General Hospital of Central Theater Command ([2018] 003–1).

### 2.2. Procedure and stimulus

**Figure 1:**
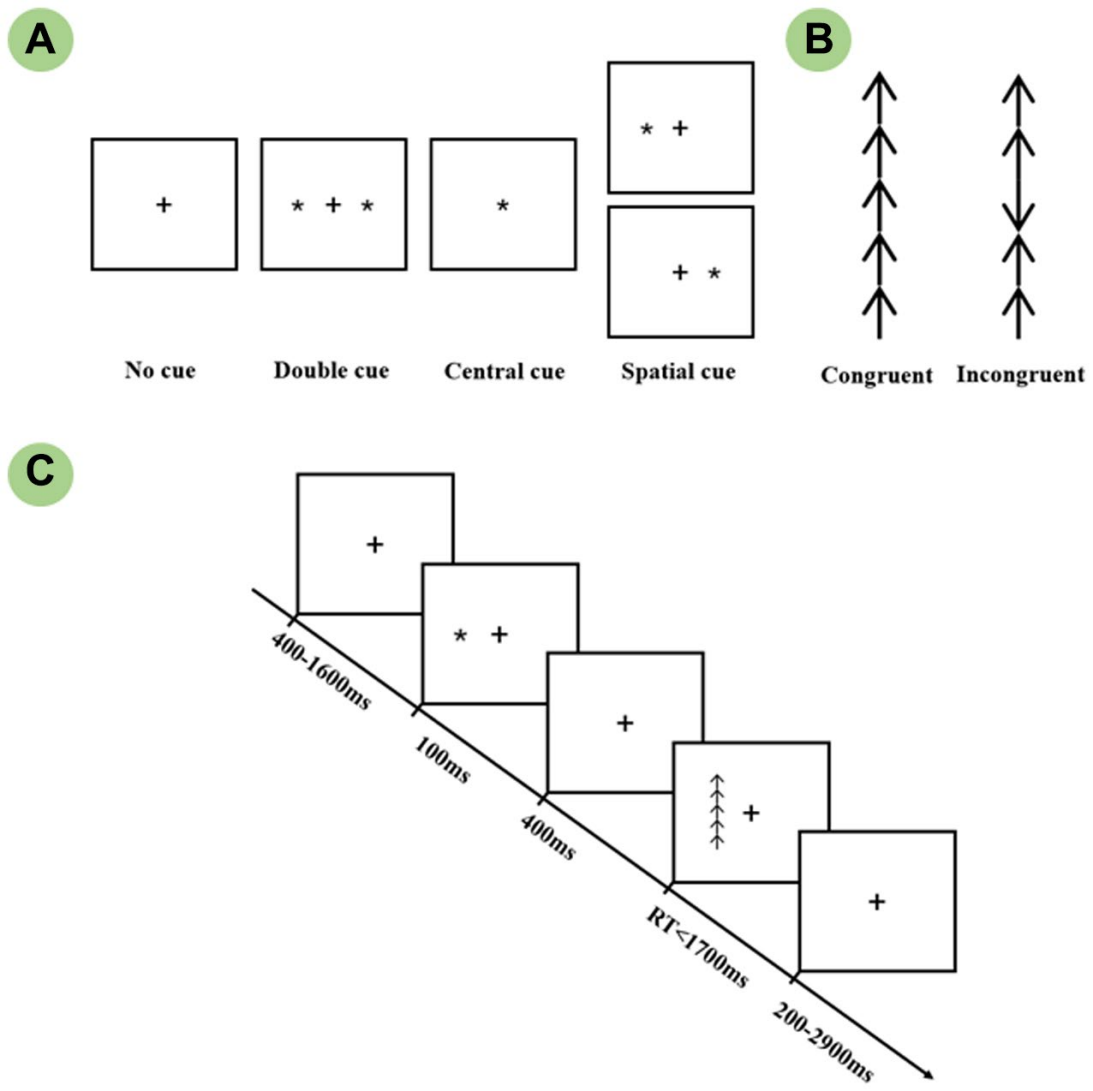
The schematic of the Lateralized Attention Network Test (LANT). **(A)** Four cue conditions: No cue, Double cue, Central cue, and Spatial cue. **(B)** Two target conditions: Congruent and Incongruent targets. **(C)** The procedure of a single trial.

The revised lateralized attention network test which was originally designed by Green et al. was conducted to measure the efficiency of attention networks within each hemisphere. Each trial, as shown in Figure 1, was first presented with a 400-1,600 ms random fixation on a white background and was followed by a 100 ms cue stimulus presented randomly. A cue-to-target interval of 400 ms was then presented to avoid the overlap between two adjacent ERP components. The target was presented up to 1700 ms until the subject made a response. A blank screen with fixation was presented at the end of the trial. Each trial lasted for 4,000 ms, and the subject was instructed to respond to the target as quickly as possible. The cue stimuli comprised five conditions: No cue, Central cue, Double cue, and Left and Right spatial cue. All spatial cues used in this study were validated, i.e., the target always appeared in the cued visual field. Each target stimulus was composed of a matrix of five arrows, the arrow array was displayed at 1.62° to the left or right of fixation, as well as 1.72° of each vertical side. The central arrow was flanked by arrows in the same direction as the target (Congruent condition), or in the opposite direction from the target (Incongruent). In comparison to the congruent conditions, the incongruent target induced stronger conflict interference, which, in turn, required more attentional resources. There was one practice block and four experimental blocks, with a 2 min break between each section. The experimental block consisted of 32 conditions: 4 warning cues (No cue, Central cue, Double cue, Spatial cue) × 2 target locations (Left or Right) × 2 flanker types (Congruent, Incongruent) × 2 target directions (Up or Down). The presentation was randomly selected, and each block contained 3 circles, for a total of 32 × 3 = 96 trials. Briefly, the whole experiment contained 4 × 96 = 384 valid recorded trials, which were divided into 16 condition combinations (4 cue × 2 flanker × 2 visual field), each combination consisted of 24 trials for the averaging of EEG epochs. Twenty-four trials covering each condition were randomly presented for practice until the accuracy reached over 90. The whole experiment took approximately 35 min in total.

The cue and target stimuli were presented using E-prime 2.0 software (Psychology Software Tools, Inc., Sharpsburg, PA, United States) on a 17-inch Dell monitor which was placed 60 cm away from the eyes. In a semi-dark, quiet room, all participants were instructed to quickly respond using the same hand’s middle or index finger. The mouse was rotated 90° and the target direction corresponded to the upward and downward buttons. Response hand was counterbalanced with a fixed order of “right–left–right–left” across four experimental blocks, which minimized the impact of motor reaction on the hemispheric attentional functions.

### 2.3. Behavioral measures

Efficiencies of hemispheric attention networks were measured by comparing reaction time (RT) and error rate (ER) across conditions. Noted that responses longer than 1700 ms or shorter than 200 ms were considered delayed responses or expectancy effects and should be excluded. Therefore, lateralized network effects were calculated using the following equations, and the error rates were calculated by the same RT formula.

(1) Right alerting effect = RT_no cue_ – RT_double cue_ (right target)
(2) Left alerting effect = RT_no cue_ – RT_double cue_ (left target)
(3) Right orienting effect = RT_central cue_ – RT_right spatial cue_ (right target)
(4) Left orienting effect = RT_central cue_ – RT_left spatial cue_ (left target)
(5) Right executive control effect = RT_incongruent flanker_ – RT_congruent flanker_ (right target)
(6) Left executive control effect = RT_incongruent flanker_ – RT_congruent flanker_ (left target)

### 2.4. EEG recording and analysis

A 64-channel EEG data was recorded by ANT Neuro’s eegoTM mylab EEG system and the electrode distribution was matched to the international 10–20 system. Online EEG acquisition was performed with eegoTM acquisition software, with a sampling rate of 1,000 Hz and an online bandpass of 0.3–100 Hz. The impedance was reduced below 10 KΩ, and all electrodes were referenced to the CPz electrode. Offline EEG data was analyzed with MATLAB 2020b platform and was performed with a bandpass of 1–40 Hz, as well as a notch filter at 50 Hz to remove alternative interferences. All EEG data were segmented from 200 ms pre-stimulus to 1,000 ms post-stimulus, and the baseline was corrected by the mean amplitude of the 200 ms pre-stimulus. Artifacts were removed by independent component analysis (Chaumon et al., 2015). EEG data were re-referenced to average reference, and EEG epochs were then separately extracted and averaged across conditions for each subject. Finally, the mean amplitude of ERP component was extracted for visualization of the topographic map and further statistical analysis.

### 2.5. Serum hormone levels assessment

In patients with pituitary adenoma, rapid peripheral venous blood samples were taken at 8:00–9:30 am to minimize the effect of hormonal circadian rhythms. Chemiluminescent immunoassays (Roche, Cobas 8,000, Switzerland) were used to determine the serum prolactin level (ng/ml). Serum was also diluted 1:100 if necessary to rule out hook effects.

### 2.6. Statistical analysis

Behavioral and EEG data were analyzed using the statistical software SPSS 27.0. The demographic characteristics of the two groups were compared by independent samples *t*-test (e.g., age, education) and Chi-square test (gender). Behavioral data were extracted and summarized for each condition, and the mean reaction time and the mean error rate of the subjects were computed. Condition-level analysis: All data were included in a 4-way repeated-measures ANOVA for further analysis. 4 Cue (No cue, Central cue, Double cue, Spatial cue) × 2 Target (Congruent, Incongruent) × 2 Visual field (Left and Right) × 2 Group (PA and HCs). Network-level analysis: A 2-way repeated-measures ANOVA (2 Visual field × 2 Group) was conducted for each network based on the network effect results.

The EEG segments were averaged separately for 4 cues and 2 target stimuli. Alerting network: The mean amplitude of N1 in the averaged left (P7) and right (P8) parietal regions was extracted and included in a 2-way repeated-measures ANOVA (2 Cue type × 2 Group). Orienting network: The same N1 component was used to measure the orienting effect. Given the cue stimuli were presented in both visual fields, the factors of visual field (LVF, RVF) and hemisphere (Left hemisphere (LH), Right hemisphere (RH)) were included in a 3-way repeated-measures ANOVA (3 Cue type × 2 hemisphere × 2 group). Executive control network: Resembling the orienting network, the visual field, and the hemisphere factors were added in a 4-way repeated-measures ANOVA (2 Target type × 2 visual field × 2 hemisphere × 2 group). Multivariate test results were examined if violations of sphericity. *Post-hoc* simple effect results were corrected by the Bonferroni approach. At the same time, a Spearman correlation analysis was performed to investigate the relationship between serum PRL levels and attentional function. α = 0.05.

## 3. Results

### 3.1. Demographic characteristics

Twenty patients with pituitary adenomas were recruited in this study, and 2 were excluded because of excessive artifacts in the EEG data. Twenty-five healthy controls were recruited, 1 was excluded due to high error rates, and 4 were excluded due to poor data quality. Ultimately, 18 patients and 20 healthy controls matched for age, sex, and education were included in this study. Detailed characteristics are shown in Table 1.

**Table 1:** Comparison of the demographic characteristics in both groups.

	HCs	Patients	*p*
*N*	20	18	/
Females/Males	8/12	12/6	0.100
Age (years) (M ± SD)	34.55 ± 11.048	38.78 ± 10.619	0.238
Education (years) (M ± SD)	14.45 ± 3.332	12.67 ± 3.531	0.118

### 3.2. Behavioral results

#### 3.2.1. Condition-level analysis

**RT:** A 2 group × 4 cue × 2 flanker ×2 visual field 4-way repeated-measures ANOVA was conducted to investigate the group differences across conditions. A main group effect was found [*F* (1,36) = 13.815, *p* = 0.001], Post-hoc analysis revealed that the reaction time in the PA group was longer than HCs. [**PA:** (713.840 ± 21.851) ms; **HCs:** (601.888 ± 20.730) ms]. Meanwhile, there was a significant main effect of cue and flanker condition [**Cue:** *F* (3,34) = 247.788, *p* < 0.001; **Flanker:** *F* (1,36) = 299.561, *p* < 0.001]. Further analysis indicated a significant group interaction effect with cue and flanker. [Cue*Group: *F* (3,34) = 2.966, *p* = 0.046; Flanker*Group: *F* (1,36) = 18.533, *p* < 0.001]. Simple analysis suggested that RTs were significantly longer in the PA group for all types of cues and flankers (*p* < 0.01).

**ER:** Resembling ANOVA was conducted, and the results revealed a significant main cue effect [*F* (3,108) = 6.264, *p* < 0.001] and main flanker effect [*F* (1,36) = 12.615, *p* = 0.001]. For both groups, the ANOVAs revealed a flanker × cue interaction effect. [*F* (3,108) = 2.736, *p* = 0.047]. In contrast, there was no significant difference between two groups [*F* (1,36) = 0.625, *p* = 0.434].

#### 3.2.2. Network-level analysis

**RT:** The ANOVA of alerting network exhibited a significant main group effect. [*F* (1,36) = 8.460, *p* = 0.006], with the HCs group showing greater network efficiency than the PA group [**PA:** (33.041 ± 4.884) ms; **HCs:** (52.624 ± 4.634) ms]. The main visual field effect was significant [*F* (1,36) = 7.003, *p* = 0.012], showing a left visual field bias in alerting network efficiency [**R:** (35.157 ± 4.528) ms; **L:** (50.507 ± 4.357) ms]. For the orienting network, both main group effect and group interaction effects were not reached significant differences. [*F* (1,36) = 0.434, *p* = 0.514]. Concerning the executive control network, a significant difference was found between the two groups [*F* (1,36) = 17.251, *p* < 0.001], with the PA group having a significantly greater efficiency. [**PA:** (104.924 ± 7.872) ms; **HCs:** (59.858 ± 7.468) ms].

**ER:** For each attentional subnetwork, no significant main effects or interaction effects were found in the ER analysis. [**Alert:** *F* (1,36) = 0.045, *p* = 0.832; **Orient:** *F* (1,36) = 0.037, *p* = 0.849; **Executive:** *F* (1,36) = 0.339, *p* = 0.564].

### 3.3. Electrophysiological results

#### 3.3.1. Alerting (no cue vs. double cue)

**Figure 2:**
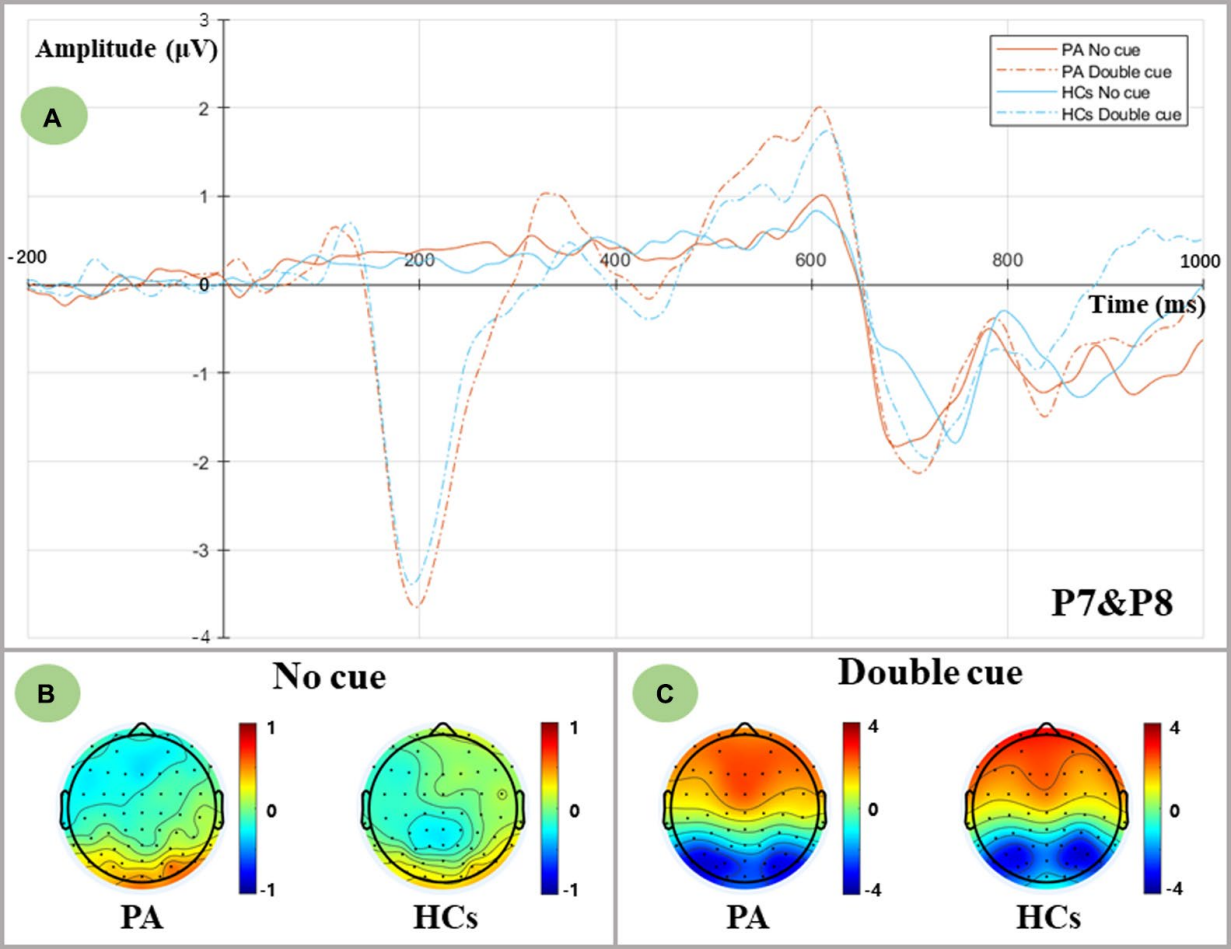
Alerting-related N1 components as measured at averaged P7 and P8 electrodes and topographic voltage maps for two groups. **(A)** Grand-averaged N1 components at averaged P7 and P8 electrodes. PA group: red lines; HCs group: blue lines; No cue condition: solid lines; Double cue condition: dashed lines. **(B)** Topographic voltage maps in the no cue condition for both groups. **(C)** Topographic voltage maps in the double cue condition for both groups.

The waveforms of ERPs evoked by no cue and double cue are depicted in Figure 2. The mean amplitudes of cue-locked N1 in the time window of 180–230 ms were averaged and extracted for both temporoparietal regions (P7, P8). Data were calculated for a 2 group (PA vs. HCs) × 2 cue (No cue vs. Double cue) repeated-measures ANOVA. Although the results indicated that both groups showed the main cue effect [*F* (1,36) = 85.585, *p* < 0.001], no group differences were found in the alerting network [*F* (1,36) = 0.115, *p* = 0.736].

#### 3.3.2. Orienting (central cue vs. spatial cue)

**Figure 3:**
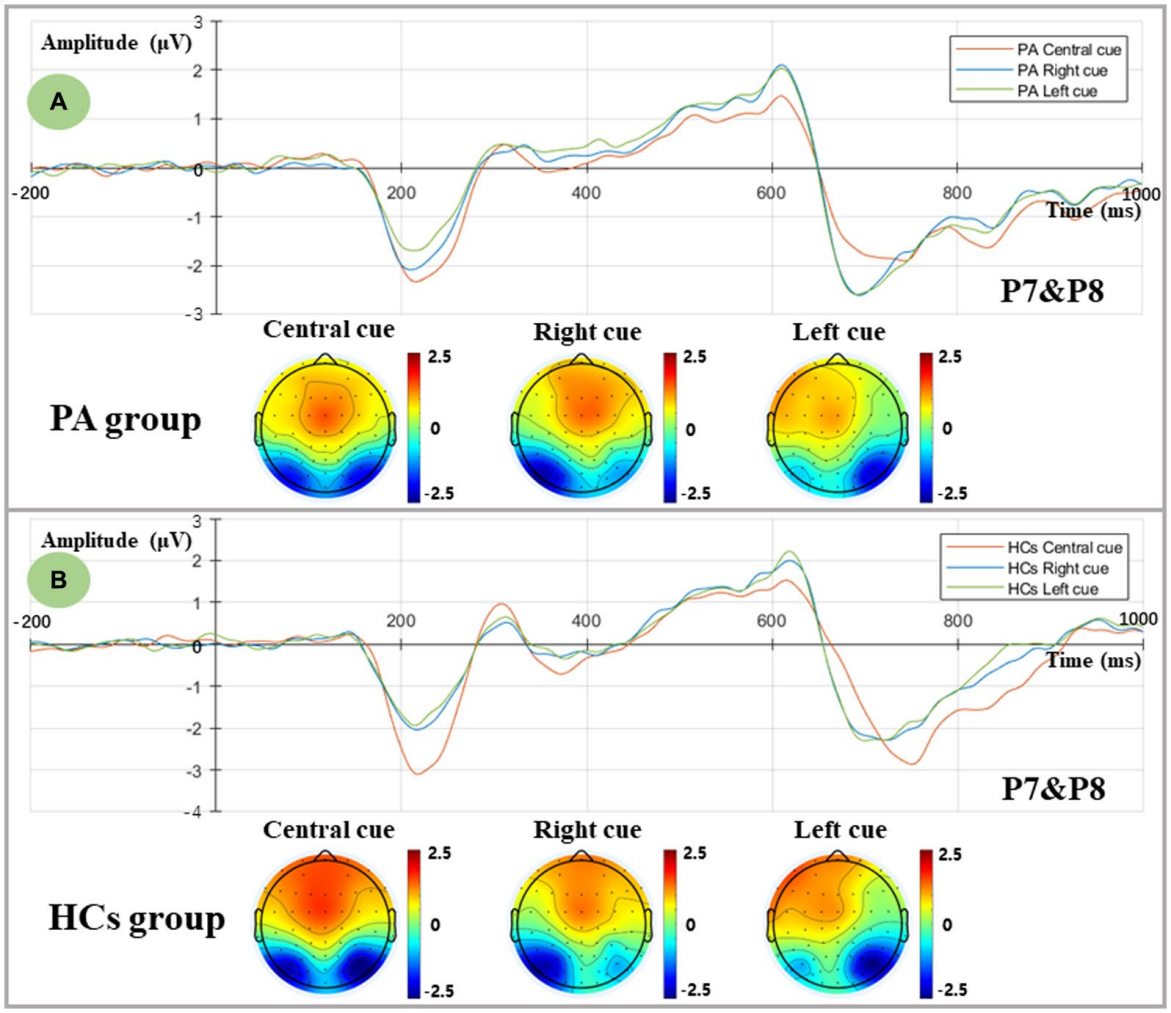
Orienting-related N1 components as measured at averaged P7 and P8 electrodes and topographic voltage maps for two groups. **(A)** Grand-averaged N1 components and topographic voltage maps in the central, right, and left spatial cue conditions for the PA group. Central cue: red line; Right spatial cue: blue line; Left spatial cue: green line. **(B)** Grand-averaged N1 components and topographic voltage maps in the central, right, and left spatial cue conditions for the HCs group. Central cue: red line; Right spatial cue: blue line; Left spatial cue: green line.

Figure 3 shows ERPs evoked by central and spatial cues in two groups. Given the lateralization of the orienting network, bilateral temporoparietal regions (Left-P7, Right-P8) were considered for extracting the mean amplitude of N1 within the defined time window of 180–230 ms. The ANOVA results showed a significant cue effect and a Cue×Hemisphere interaction [**Cue:** *F* (2,35) = 13.493, *p* < 0.001; Cue*Hemisphere: *F* (2,35) = 70.476, *p* < 0.001]. Post-hoc simple effect analysis revealed a larger N1 amplitude in the central cue compared to the two spatial cues [Central cue: (−2.122 ± 0.311) μV; Right cue: (−1.643 ± 0.243) μV; Left cue: (−1.442 ± 0.245) μV]. Regarding the interaction effects, in the central cue condition, a similar distribution of N1 was found in both left and right hemispheres [*F* (1,36) = 0.693, *p* = 0.411], whereas the unilateral visual field orienting response (spatial cue) elicited contralateral parietal activation [**Right cue:** *F* (1,36) = 42.566, *p* < 0.001; **Left cue:** *F* (1,36) = 42.565, *p* < 0.001]. In contrast, no group effect was found in the orienting network [*F* (1,36) = 0.017, *p* = 0.898], suggesting that comparable orienting functions were elicited in both groups.

#### 3.3.3. Executive control (incongruent vs. congruent)

**Figure 4:**
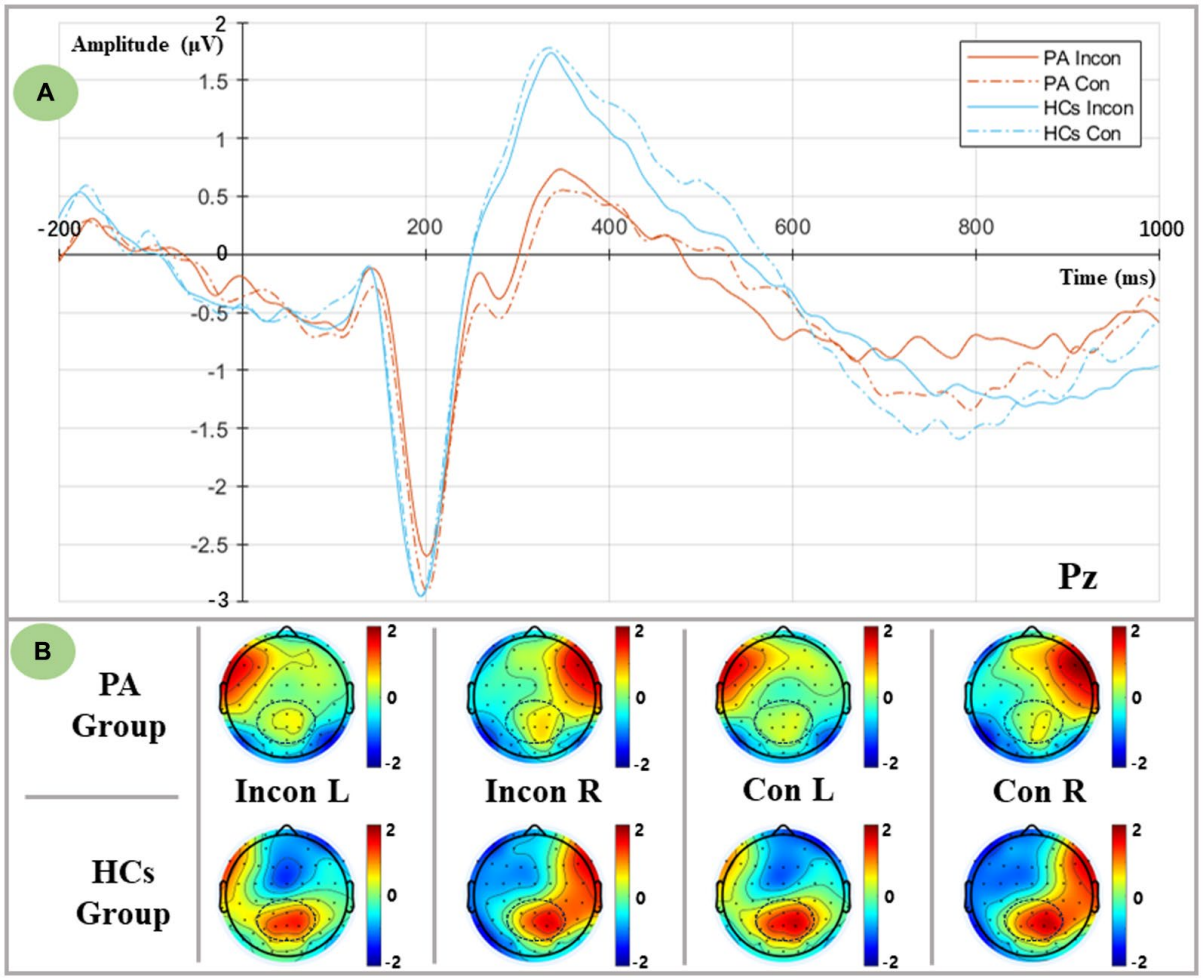
Executive-related P3 components as measured at the Pz electrode, and lateralized topographic voltage maps between groups and conditions. **(A)** Grand averaged P3 components from the Pz electrode in both flanker conditions. PA group: red lines; HCs group: blue lines; Incongruent condition: solid lines; Congruent condition: dashed lines. **(B)** Lateralized topographic voltage maps between two groups in (1) Incongruent left spatial condition, (2) Incongruent right spatial condition, (3) Congruent left spatial condition, and (4) Congruent right spatial condition.

The incongruent and congruent conditions are shown in Figure 4. We defined the time window of P3 as 290–400 ms based on the peak latency. To further examine the distribution of P3 in both hemispheres, P1 and P3 were categorized as the left central parietal area (LCP), and P2 and P4 were categorized as the right central parietal area (RCP). The ANOVA results indicated that there was a significant main group effect and a flanker×group interaction [**Group:** *F* (1,36) = 6.854, *p* = 0.013; Flanker*Group: *F* (1,36) = 5.283, *p* = 0.027]. Post-hoc analysis revealed that the P3 amplitude of the HCs was larger than that of the PA [HCs: (1.179 ± 0.284) μV; PA: (0.098 ± 0.300) μV], and both flanker conditions exhibited an increase in the amplitude of P3 in the HCs compared to the PA group [**Incon:** *F* (1,36) = 4.796, *p* = 0.035; **Con:** *F* (1,36) = 9.065, *p* = 0.005]. Notably, a significant main effect of the hemisphere has been observed in the ANOVA results and interacted with the visual field [**Hemisphere:** *F* (1,36) = 7.162, *p* = 0.011; VF*Hemisphere: *F* (1,36) = 11.254, *p* = 0.002]. Simple effect analysis showed significant lateralization of the right hemispheric which indicated a larger P3 amplitude in the RCP compared with the LCP [**LH:** (0.426 ± 0.229) μV; **RH:** (0.851 ± 0.213) μV]. Moreover, no difference in P3 distribution was observed when the target presented in the LVF [*F* (1,36) = 0.091, *p* = 0.764], while significant RCP-biased P3 distribution was observed when the target presented in the RVF [*F* (1,36) = 18.479, *p* < 0.001], showing an asymmetric distribution of P3.

Furthermore, a significant main group effect was found in the flanker×VF × hemisphere interaction [*F* (1,36) = 7.544, *p* = 0.009], and a further simple effect analysis indicated that regardless of the flanker conditions and the different visual fields, HCs elicited a larger mean P3 amplitude in the RCP compared to the PA group (*p* < 0.05). Considering the incongruent right visual field, a consistent RCP dominance of the P3 was found in the HCs [*F* (1,36) = 16.431, *p* < 0.001], whereas a uniform distribution of P3 was found in the PA group [*F* (1,36) = 3.669, *p* = 0.063].

### 3.4. Correlation analysis results

**Figure 5:**
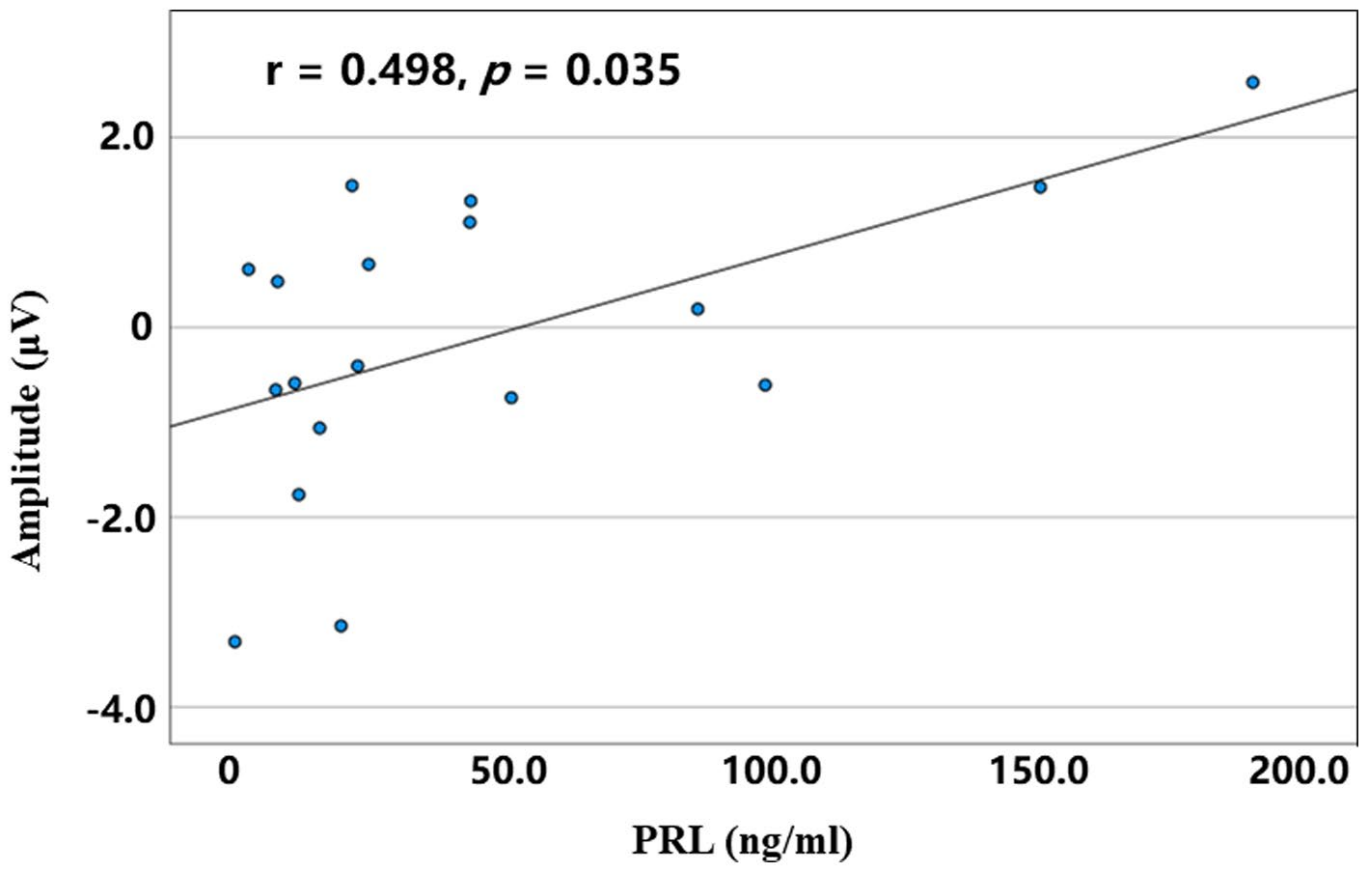
The correlation between serum PRL level and mean amplitudes of the left central parietal P3 in the incongruent right spatial condition for the PA group.

In the PA group, a bivariate Spearman correlation analysis was performed between the serum PRL level and mean amplitude of P3 across conditions. The results revealed that a positive correlation was observed between the serum PRL level and the P3 amplitude of the LCP in the incongruent RVF conditions (r = 0.498, *p* = 0.035; Figure 5).

### 3.5. Exploration results

Behavioral findings depicted increased efficiency of the executive control network in the PA group. Reversed P3 results were demonstrated, suggesting executive dysfunction in the PA group. Thus, to resolve the conflict results, we conducted another 2 visual field × 2 flanker × 2 group, 3-way repeated-measures ANOVA. The results revealed a main group effect and a significant flanker×group interaction [**Group:** *F* (1,36) = 14.671, *p* < 0.001; Flanker*Group: *F* (1,36) = 17.251, *p* < 0.001]. Simple effect analysis results exhibited a prolonged RT in the PA group [**PA:** (716.488 ± 21.729) ms; **HCs:** (601.762 ± 20.614) ms]. Furthermore, PA patients responded more slowly than HCs in both flanker conditions [**Incon:** *F* (1,36) = 18.902, *p* < 0.001; **Con:** *F* (1,36) = 9.925, *p* = 0.003]. In contrast, the target effect size (RT_incon_ - RT_con_) was significantly larger in the PA group relative to the HCs group [**PA:** (104.924 ± 7.872) ms; **HCs:** (59.858 ± 7.468) ms].

## 4. Discussion

We combined a lateralized attention network paradigm with event-related potential techniques to examine the impairment and lateralization of each attention subnetwork in pituitary adenoma patients. In terms of behavioral performance, the temporal and spatial cue effects, as well as the flanker effect, could be evoked significantly by the LANT task. In line with previous findings based on ANT and LANT paradigms (Greene et al., 2008; Neuhaus et al., 2010; Thiebaut de Schotten et al., 2011), the decreased reaction time reflected the facilitatory effect of temporal and spatial cues on target responses. Both groups exhibited similar error rates in each subnetwork, but the PA group showed prolonged RTs in all conditions relative to HCs. To maintain relatively high accuracy for the same cue and target stimuli, excessed attentional resources have to pay for conflict resolution. Thus, we believed that the pituitary adenoma was more likely to impair global attentional processing, which has been demonstrated in previous studies (Chen et al., 2022).

Network efficiency results revealed that the PA group had lower alerting network efficiency but higher executive control network efficiency relative to the HCs. Given the differences in the visual field, a significant LVF alerting effect was found in the PA group. Although some studies have shown a significant LVF-RH dominance for alerting (Heilman and Van Den Abell, 1979; Funnell et al., 2003), these warning cues were only presented on the unilateral visual field instead of bilateral visual fields. In line with the present study, the results of Asanowicz (Asanowicz et al., 2012) indicated no visual field differences in the alerting network efficiency, which was induced by a revised LANT paradigm. Therefore, the visual field differences in alerting network in the PA group could be explained by the small sample size and the heterogeneity of tumor types. Regarding the inconsistent alerting network results in the N1, we hypothesized that RT was an indicator that measured alerting indirectly, Because RT reflected the full stages of cognition from receiving warning signals, arousing the alerting state to making a response ultimately. ERPs, on the other hand, can capture and quantify the alerting state specifically, and may not be predicted accurately by the behavioral results.

Although greater executive control efficiency was found in the PA group compared to HCs, further results from the ANOVA revealed that the PA group had prolonged RTs in both flanker conditions, and the difference was still greater than the HCs. Whereas the PA group responded slower in both target conditions, and significantly longer RTs were observed in the PA group when an incongruent condition was presented. Thus, we believed that the smaller efficiency of the executive control network indicated greater executive functions, which was consistent with previous evidence explained by the right hemisphere specialization in executive functions (Milham et al., 2001; Asanowicz et al., 2012). Overall, we hypothesized that greater efficiency of the executive control network corresponded to worse executive functions, indicating dysfunction of inhibition control in the PA group.

P3 modulated the inhibition control processes to the target stimuli, as well as the allocation of attentional resources (Polich, 2007; Kratz et al., 2011). In the HCs, the direction of P3 evoked by the target was opposite to RT, with larger P3 amplitudes corresponding to shorter RTs, which may be interpreted as task difficulty (Polich, 1987). More difficult tasks required greater attentional demands and evoked smaller P3 components, in other words, decreased P3 components mirrored the limitation of attentional capacity. Therefore, reduced P3 amplitudes across conditions in the PA group were indicative of executive control dysfunction and decreased attention allocation, which was supported by previous findings in PA patients (Tooze et al., 2009). The LANT task provided a notable advantage for investigating the lateralization of attention networks. The stimuli in the ANT task were presented on the midline, thus no visual field differences were induced, whereas the stimuli in the LANT task were presented in both visual fields and might be induced by the hemispheric differences in the P3 component. Studies have found a right hemisphere dominance in executive functions, whereas the left hemisphere has been shown to play an important role in semantic information processing (MacLeod, 1991; Russell-Giller et al., 2021). Therefore, the RCP distribution of the P3 component in the present study supported the RH dominance theory of executive function. Furthermore, the ANOVA results also exhibited a significant VF × hemisphere interaction, which indicated that the RH dominated the bilateral visual field information, while the LH only predominantly processed the left visual field information. The interaction results partially supported the theory of hemispheric lateralization (Nobre et al., 1997). Indeed, studies with a rapid serial visual presentation (RSVP) task have demonstrated that larger RH P3 was evoked in the RVF, whereas the LVF predominantly evoked the P3 in the LH, indicating a significant ipsilateral activation (Zhang et al., 2022). Further efforts at lateralization were needed to interpret the potential VF asymmetry of the executive control network.

Surprisingly, the 4-way (Flanker×VF × Hemisphere×Group) ANOVA ERPs results showed a main group effect in the pattern of hemispheric asymmetry when the flanker factor was included. Unlike the HCs group with a right hemispheric dominance when an incongruent target presented on the RVF, the PA group manifested a bilateral distribution of the P3. One reasonable interpretation was that reduced RCP capacity to RVF stimuli led to a compensatory activation in the LCP, highlighting the recruitment of resources from the non-task-dominant hemisphere. This view was consistent with Paitel’s findings for age-related alteration in inhibitory control, whereas compensatory recruitment occurred at low-moderate task demands, which was indicative of depleted neural reserves (Paitel and Nielson, 2021). However, different from the stop-signal task (SST), the P3 component evoked by LANT was correlated with the allocation of attentional resources and conflict resolution. Therefore, under high attentional demand, additional contralateral hemispheric resources of attention were recruited for task response, leading to enhanced activation in the LCP and the absence of RH dominance. In Weissman and Welcome’s view (Weissman and Banich, 2000; Welcome and Chiarello, 2008), increasing task difficulty required the recruitment of both hemispheres, resulting in an attenuated hemispheric asymmetry. Overall, to overcome the decreased attentional function in the RCP under high conflict conditions, attentional resources in the LCP were required in patients with pituitary adenomas, which led to the elimination of the RH-dominated pattern.

This pattern of contralateral activation in the orienting network was consistent with previous evidence (Hill-Jarrett et al., 2015). Unfortunately, however, the pattern of N1 activation was similar in both groups, suggesting that PA patients may preserve adequate orienting function. Retinal eccentricity modulated attentional demands (Beaton and Blakemore, 1981; Asanowicz et al., 2012), and larger eccentricities may make targets difficult to discriminate and require additional attentional resources. Therefore, in the present study, cues and target stimuli may not be presented peripherally enough for allocating resources in orienting. The greater horizontal distance of stimuli presentation and increased differentiation of orienting networks between groups may be beneficial for the further investigation in functional status of orienting networks in PA patients.

Lastly, a significant negative correlation between serum PRL levels and executive functions was found in prolactinoma patients, suggesting the toxic effect of hyperprolactinemia on cognitive functions (Yao et al., 2017; Chen et al., 2022). Meanwhile, the absence of lateralization in the PA group may result from compound factors that mainly contained the compensatory effect of LH and the destructive effect of hyperprolactinemia. The former enhanced the recruitment of attentional resources, and the latter may attenuate the standard lateralized activation pattern and reduce the efficiency of the executive control network.

## 5. Conclusion

The present study provided behavioral and electrophysiological evidence of alteration in lateralized attention networks evoked by the LANT paradigm in patients with pituitary adenoma. No ERP differences were found in alerting and orienting network, except for a specialized pattern of contralateral activation in orienting N1 component. More importantly, the executive network P3 exhibited lateralization to RH, and decreased P3 amplitude in the PA group revealed impairment of inhibition control and reduced attentional resource allocation. Moreover, attenuated hemispheric asymmetry of P3 was observed in PA patients, which may be attributed to the mixed effect including the compensatory recruitment of attentional resources in LCP and the destructive effects of hyperprolactinemia. These findings suggested that, in the lateralized condition, the decreased P3 in the RCP and the diminished hemispheric asymmetry under high conflict load, may serve as the potential biomarkers of attentional dysfunction in patients with pituitary adenoma. In addition, the findings above provide further evidence for functional recovery in post-surgery pituitary adenoma patients.

## Data availability statement

The original contributions presented in the study are included in the article/supplementary material, further inquiries can be directed to the corresponding authors.

## Ethics statement

The studies involving human participants were reviewed and approved by the Ethics Committee of the General Hospital of Central Theater Command. Written informed consent to participate in this study was provided by the participants’ legal guardian/next of kin.

## Author contributions

SW proposed and performed the present study, and contributed to collecting and analyzing the experimental data, as well as writing the manuscript. ZF reviewed the manuscript and suggested article revisions. YS contributed to collecting data and writing code. MZ provided clinical guidance. AC and CC contributed to designing the experiment. JS supervised and funded the present study. All authors contributed to the article and approved the submitted version.

## Funding

The funding of the present study was provided by the National Natural Science Foundation of China (81870863).

## Conflict of interest

The authors declare that the research was conducted in the absence of any commercial or financial relationships that could be construed as a potential conflict of interest.

## Publisher’s note

All claims expressed in this article are solely those of the authors and do not necessarily represent those of their affiliated organizations, or those of the publisher, the editors and the reviewers. Any product that may be evaluated in this article, or claim that may be made by its manufacturer, is not guaranteed or endorsed by the publisher.

## References

[ref1] Adólfsdóttir S. Sørensen L. Lundervold A. J. (2008). The attention network test: a characteristic pattern of deficits in children with ADHD. Behav. Brain Funct. 4:9. doi: 10.1186/1744-9081-4-9, PMID: 18269768 PMC2265730

[ref2] Asanowicz D. Marzecová A. Jaśkowski P. Wolski P. (2012). Hemispheric asymmetry in the efficiency of attentional networks. Brain Cogn. 79, 117–128. doi: 10.1016/j.bandc.2012.02.014, PMID: 22475579

[ref3] Beaton A. Blakemore C. (1981). Orientation selectivity of the human visual system as a function of retinal eccentricity and visual hemifield. Perception 10, 273–282. doi: 10.1068/p100273, PMID: 7329749

[ref4] Brooks J. L. Della Sala S. Darling S. (2014). Representational pseudoneglect: a review. Neuropsychol. Rev. 24, 148–165. doi: 10.1007/s11065-013-9245-2, PMID: 24414221

[ref5] Butterbrod E. Gehring K. Voormolen E. H. Depauw P. Nieuwlaat W. A. Rutten G. M.. (2019). Cognitive functioning in patients with nonfunctioning pituitary adenoma before and after endoscopic endonasal transsphenoidal surgery. J. Neurosurg. 133, 709–716. doi: 10.3171/2019.5.Jns19595, PMID: 31443073

[ref6] Cao S. Zhang J. Wang Z. Pan W. Tian Y. Hu P.. (2020). Laterality of Attentional networks in patients with cerebral small vessel disease. Front. Aging Neurosci. 12:21. doi: 10.3389/fnagi.2020.00021, PMID: 32265683 PMC7098913

[ref7] Chaumon M. Bishop D. V. Busch N. A. (2015). A practical guide to the selection of independent components of the electroencephalogram for artifact correction. J. Neurosci. Methods 250, 47–63. doi: 10.1016/j.jneumeth.2015.02.025, PMID: 25791012

[ref8] Chen A. Cao C. Liu B. Wang S. Wu S. Xu G.. (2022). Hyperprolactinemia associated with Attentional processing and interference control impairments in patients with Prolactinomas. Brain Sci. 12:1091. doi: 10.3390/brainsci12081091, PMID: 36009154 PMC9406026

[ref9] Chen A. Zhang Z. Cao C. Lu J. Wu S. Ma S.. (2021). Altered attention network in paratroopers exposed to repetitive subconcussion: evidence based on behavioral and event-related potential results. J. Neurotrauma 38, 3306–3314. doi: 10.1089/neu.2021.0253, PMID: 34549595

[ref10] de Oliveira C. Naliato E. Dutra Violante A. H. Caldas D. Lamounier Filho A. Rezende Loureiro C.. (2008). Quality of life in women with microprolactinoma treated with dopamine agonists. Pituitary 11, 247–254. doi: 10.1007/s11102-008-0091-9, PMID: 18270842

[ref11] Fan J. McCandliss B. D. Fossella J. Flombaum J. I. Posner M. I. (2005). The activation of attentional networks. NeuroImage 26, 471–479. doi: 10.1016/j.neuroimage.2005.02.004 15907304

[ref12] Fan J. McCandliss B. D. Sommer T. Raz A. Posner M. I. (2002). Testing the efficiency and independence of attentional networks. J. Cogn. Neurosci. 14, 340–347. doi: 10.1162/089892902317361886, PMID: 11970796

[ref13] Funnell M. G. Corballis P. M. Gazzaniga M. S. (2003). Temporal discrimination in the split brain. Brain Cogn. 53, 218–222. doi: 10.1016/s0278-2626(03)00113-1, PMID: 14607151

[ref14] Greene D. J. Barnea A. Herzberg K. Rassis A. Neta M. Raz A.. (2008). Measuring attention in the hemispheres: the lateralized attention network test (LANT). Brain Cogn. 66, 21–31. doi: 10.1016/j.bandc.2007.05.003, PMID: 17590491 PMC4283820

[ref15] Hauser B. M. Lau A. Gupta S. Bi W. L. Dunn I. F. (2019). The Epigenomics of pituitary adenoma. Front Endocrinol (Lausanne) 10:290. doi: 10.3389/fendo.2019.00290, PMID: 31139150 PMC6527758

[ref16] Heilman K. M. Van Den Abell T. (1979). Right hemispheric dominance for mediating cerebral activation. Neuropsychologia 17, 315–321. doi: 10.1016/0028-3932(79)90077-0, PMID: 514469

[ref17] Hill-Jarrett T. G. Gravano J. T. Sozda C. N. Perlstein W. M. (2015). Visuospatial attention after traumatic brain injury: the role of hemispheric specialization. Brain Inj. 29, 1617–1629. doi: 10.3109/02699052.2015.1075155, PMID: 26451899

[ref18] Kaufman D. A. Sozda C. N. Dotson V. M. Perlstein W. M. (2016). An event-related potential investigation of the effects of age on alerting, orienting, and executive function. Front. Aging Neurosci. 8:99. doi: 10.3389/fnagi.2016.00099, PMID: 27242511 PMC4860424

[ref19] Kratz O. Studer P. Malcherek S. Erbe K. Moll G. H. Heinrich H. (2011). Attentional processes in children with ADHD: an event-related potential study using the attention network test. Int. J. Psychophysiol. 81, 82–90. doi: 10.1016/j.ijpsycho.2011.05.008, PMID: 21641942

[ref20] Lundervold A. J. Adolfsdottir S. Halleland H. Halmøy A. Plessen K. Haavik J. (2011). Attention network test in adults with ADHD--the impact of affective fluctuations. Behav. Brain Funct. 7:27. doi: 10.1186/1744-9081-7-27, PMID: 21794128 PMC3168400

[ref21] MacLeod C. M. (1991). Half a century of research on the Stroop effect: an integrative review. Psychol. Bull. 109, 163–203. doi: 10.1037/0033-2909.109.2.163, PMID: 2034749

[ref22] Melmed S. (2020). Pituitary-tumor Endocrinopathies. N. Engl. J. Med. 382, 937–950. doi: 10.1056/NEJMra1810772 32130815

[ref23] Mesulam M. M. (1999). Spatial attention and neglect: parietal, frontal and cingulate contributions to the mental representation and attentional targeting of salient extrapersonal events. Philos. Trans. R. Soc. Lond. Ser. B Biol. Sci. 354, 1325–1346. doi: 10.1098/rstb.1999.0482, PMID: 10466154 PMC1692628

[ref24] Milham M. P. Banich M. T. Webb A. Barad V. Cohen N. J. Wszalek T.. (2001). The relative involvement of anterior cingulate and prefrontal cortex in attentional control depends on nature of conflict. Brain Res. Cogn. Brain Res. 12, 467–473. doi: 10.1016/s0926-6410(01)00076-3, PMID: 11689307

[ref25] Müssig K. Besemer B. Saur R. Klingberg S. Häring H. U. Gallwitz B.. (2011). Deteriorated executive functions in patients with successful surgery for pituitary adenomas compared with other chronically ill patients. J. Int. Neuropsychol. Soc. 17, 369–375. doi: 10.1017/s1355617710001645 21205414

[ref26] Neuhaus A. H. Urbanek C. Opgen-Rhein C. Hahn E. Ta T. M. Koehler S.. (2010). Event-related potentials associated with attention network test. Int. J. Psychophysiol. 76, 72–79. doi: 10.1016/j.ijpsycho.2010.02.005 20184924

[ref27] Nobre A. C. Sebestyen G. N. Gitelman D. R. Mesulam M. M. Frackowiak R. S. Frith C. D. (1997). Functional localization of the system for visuospatial attention using positron emission tomography. Brain 120, 515–533. doi: 10.1093/brain/120.3.515, PMID: 9126062

[ref28] Paitel E. R. Nielson K. A. (2021). Temporal dynamics of event-related potentials during inhibitory control characterize age-related neural compensation. Symmetry (Basel) 13:2323. doi: 10.3390/sym13122323, PMID: 35923222 PMC9345327

[ref29] Peace K. A. Orme S. M. Padayatty S. J. Godfrey H. P. Belchetz P. E. (1998). Cognitive dysfunction in patients with pituitary tumour who have been treated with transfrontal or transsphenoidal surgery or medication. Clin. Endocrinol. 49, 391–396. doi: 10.1046/j.1365-2265.1998.00543.x 9861332

[ref30] Peace K. A. Orme S. M. Thompson A. R. Padayatty S. Ellis A. W. Belchetz P. E. (1997). Cognitive dysfunction in patients treated for pituitary tumours. J. Clin. Exp. Neuropsychol. 19, 1–6. doi: 10.1080/01688639708403831 9071636

[ref31] Pertichetti M. Serioli S. Belotti F. Mattavelli D. Schreiber A. Cappelli C.. (2020). Pituitary adenomas and neuropsychological status: a systematic literature review. Neurosurg. Rev. 43, 1065–1078. doi: 10.1007/s10143-019-01134-z, PMID: 31250149

[ref32] Petersen S. E. Posner M. I. (2012). The attention system of the human brain: 20 years after. Annu. Rev. Neurosci. 35, 73–89. doi: 10.1146/annurev-neuro-062111-150525, PMID: 22524787 PMC3413263

[ref33] Polich J. (1987). Task difficulty, probability, and inter-stimulus interval as determinants of P300 from auditory stimuli. Electroencephalogr. Clin. Neurophysiol. 68, 311–320. doi: 10.1016/0168-5597(87)90052-9, PMID: 2439311

[ref34] Polich J. (2004). Clinical application of the P300 event-related brain potential. Phys. Med. Rehabil. Clin. N. Am. 15, 133–161. doi: 10.1016/s1047-9651(03)00109-8 15029903

[ref35] Polich J. (2007). Updating P300: an integrative theory of P3a and P3b. Clin. Neurophysiol. 118, 2128–2148. doi: 10.1016/j.clinph.2007.04.019, PMID: 17573239 PMC2715154

[ref36] Posner M. I. Petersen S. E. (1990). The attention system of the human brain. Annu. Rev. Neurosci. 13, 25–42. doi: 10.1146/annurev.ne.13.030190.000325 2183676

[ref37] Posner M. I. Rothbart M. K. Ghassemzadeh H. (2019). Restoring attention networks. Yale J. Biol. Med. 92, 139–143. PMID: 30923481 PMC6430178

[ref38] Psaras T. Milian M. Hattermann V. Gerlach C. Honegger J. (2011). Executive functions recover earlier than episodic memory after microsurgical transsphenoidal resection of pituitary tumors in adult patients – a longitudinal study. J. Clin. Neurosci. 18, 1340–1345. doi: 10.1016/j.jocn.2011.01.027, PMID: 21782447

[ref39] Russell-Giller S. Wu T. Spagna A. Dhamoon M. Hao Q. Fan J. (2021). Impact of unilateral stroke on right hemisphere superiority in executive control. Neuropsychologia 150:107693. doi: 10.1016/j.neuropsychologia.2020.107693, PMID: 33238172

[ref40] Thiebaut de Schotten M. Dell’Acqua F. Forkel S. J. Simmons A. Vergani F. Murphy D. G.. (2011). A lateralized brain network for visuospatial attention. Nat. Neurosci. 14, 1245–1246. doi: 10.1038/nn.2905 21926985

[ref41] Tooze A. Gittoes N. J. Jones C. A. Toogood A. A. (2009). Neurocognitive consequences of surgery and radiotherapy for tumours of the pituitary. Clin. Endocrinol. 70, 503–511. doi: 10.1111/j.1365-2265.2008.03464.x 19178526

[ref42] Tooze A. Sheehan J. P. (2018). Neurocognitive changes in pituitary adenoma patients after gamma knife radiosurgery. J. Neurosurg. 129, 55–62. doi: 10.3171/2018.7.Gks181595, PMID: 30544290

[ref43] Weissman D. H. Banich M. T. (2000). The cerebral hemispheres cooperate to perform complex but not simple tasks. Neuropsychology 14, 41–59. doi: 10.1037//0894-4105.14.1.41 10674797

[ref44] Welcome S. E. Chiarello C. (2008). How dynamic is interhemispheric interaction? Effects of task switching on the across-hemisphere advantage. Brain Cogn. 67, 69–75. doi: 10.1016/j.bandc.2007.11.005, PMID: 18206285 PMC2486493

[ref45] Williams R. S. Biel A. L. Wegier P. Lapp L. K. Dyson B. J. Spaniol J. (2016). Age differences in the attention network test: evidence from behavior and event-related potentials. Brain Cogn. 102, 65–79. doi: 10.1016/j.bandc.2015.12.007, PMID: 26760449

[ref46] Yao S. Song J. Gao J. Lin P. Yang M. Zahid K. R.. (2017). Cognitive function and serum hormone levels are associated with gray matter volume decline in female patients with Prolactinomas. Front. Neurol. 8:742. doi: 10.3389/fneur.2017.00742, PMID: 29434564 PMC5797301

[ref47] Zhang S. Chen X. Wang Y. Liu B. Gao X. (2022). Visual field inhomogeneous in brain-computer interfaces based on rapid serial visual presentation. J. Neural Eng. 19:016015. doi: 10.1088/1741-2552/ac4a3e, PMID: 35016160

